# A Comprehensive Identification and Function Analysis of Serine/Arginine-Rich (SR) Proteins in Cotton (*Gossypium* spp.)

**DOI:** 10.3390/ijms23094566

**Published:** 2022-04-20

**Authors:** Fei Wei, Pengyun Chen, Hongliang Jian, Lu Sun, Xiaoyan Lv, Hengling Wei, Hantao Wang, Tingli Hu, Liang Ma, Xiaokang Fu, Jianhua Lu, Shiyun Li, Shuxun Yu

**Affiliations:** 1 Zhengzhou Research Base, State Key Laboratory of Cotton Biology, Zhengzhou University, Zhengzhou 450001, China; weifei888000@outlook.com; 2 State Key Laboratory of Cotton Biology, Institute of Cotton Research of CAAS, Anyang 455000, China; arpengyun@163.com (P.C.); hongliang1102@163.com (H.J.); lvxiaoyan368@163.com (X.L.); henglingwei@163.com (H.W.); w.wanghantao@163.com (H.W.); htlgryx@163.com (T.H.); maliang3417@163.com (L.M.); xkang_2010@163.com (X.F.); lujh212760@163.com (J.L.); 3 Handan Academy of Agricultural Sciences, Handan 056001, China; sunlu1217.ok@163.com

**Keywords:** *Gossypium hirsutum*, serine/arginine-rich proteins, salt stress

## Abstract

As one of the most important factors in alternative splicing (AS) events, serine/arginine-rich (SR) proteins not only participate in the growth and development of plants but also play pivotal roles in abiotic stresses. However, the research about SR proteins in cotton is still lacking. In this study, we performed an extensive comparative analysis of SR proteins and determined their phylogeny in the plant lineage. A total of 169 SR family members were identified from four *Gossypium* species, and these genes could be divided into eight distinct subfamilies. The domain, motif distribution and gene structure of cotton SR proteins are conserved within each subfamily. The expansion of SR genes is mainly contributed by WGD and allopolyploidization events in cotton. The selection pressure analysis showed that all the paralogous gene pairs were under purifying selection pressure. Many *cis*-elements responding to abiotic stress and phytohormones were identified in the upstream sequences of the GhSR genes. Expression profiling suggested that some GhSR genes may involve in the pathways of plant resistance to abiotic stresses. The WGCNA analysis showed that *GhSCL-8* co-expressed with many abiotic responding related genes in a salt-responding network. The Y2H assays showed that *GhSCL-8* could interact with GhSRs in other subfamilies. The subcellular location analysis showed that *GhSCL-8* is expressed in the nucleus. The further VIGS assays showed that the silencing of *GhSCL-8* could decrease salt tolerance in cotton. These results expand our knowledge of the evolution of the SR gene family in plants, and they will also contribute to the elucidation of the biological functions of SR genes in the future.

## 1. Introduction

As an essential regulatory mechanism in plants, by producing protein variants with altered function, alternative splicing (AS) can significantly alter the coding capacity as well as modify gene expression by modulating transcription elongation and translation efficiency [1]. AS events play an important role in the development and response to the environment in plants [2,3,4].

In the process of AS events, serine/arginine-rich (SR) proteins play a key role. Since the first SR protein was identified in 1990 in metazoans, SR proteins have been widely studied in both animals and plants [5,6]. Typical SR proteins include one or two N-terminal RRM domains (PF00076) followed by a downstream RS domain of at least 50 amino acids with 40% RS content characterized by consecutive RS or SR repeats [7,8]. Typical SR proteins can be classified into six subfamilies, including SC, SCL, RS, SR, RSZ, and RS2Z, and three of these subfamilies (SR, SCL, and RSZ) are specific to plants [9]. The members of the SC subfamily contain a single RRM domain followed by an RS domain, whereas SCL (SC-like) subfamily members contain a short N-terminal extension that includes multiple RS and SR dipeptides. The SR subfamily contains two RRM domains, and the second one contains a conserved SWQDLKD motif. The RSZ subfamily contains a single RRM domain as well as a single zinc knuckle (ZnK) domain, whereas the members of the RS2Z subfamily contain one more ZnK domain [9]. Moreover, there are two kinds of untypical SR proteins identified in the previous studies, which are SR45a and SR45. *SR45a* encodes a protein with RS and RRM domains, whereas SR45 has two distinct RS domains located on either side of the RRM [10,11].

Previous studies have shown that SR proteins widely participate in the growth and development of plants. *SC35* and *SCL* proteins regulate the flowering time as well as the development of leaves, root length, and silique phyllotaxy in *Arabidopsis* [12]. The overexpression of *SR30* affects the splicing and growth of plants, resulting in late flowering in *Arabidopsis* [13]. In addition, the overexpression of *RS2Z33* in *Arabidopsis* leads to an increased number of embryos, thicker hypocotyl and cotyledons, altered shapes of root hairs, and elevated cell size [14].

On the other hand, SR proteins alter the tolerance to abiotic stress of plants. Heterologous expression of *Manihot esculenta* gene *MeSCL30A* in *Arabidopsis* induces hypersensitive to salt and drought stress and early flower [15]. Similarly, the overexpression of *PtSCL30* in *Arabidopsis* decreased freezing and salt tolerance [16]. *SR45* not only positively regulates salt tolerance but also changes the expression and splicing of genes related to salt stress in *Arabidopsis* [17]. In rice, *OsFKBP20-1b* interacts with *OsSR45* and adapts to abiotic stress by regulating RNA processing [18]. Moreover, *SR45a* directly interacted with the *CBP20*, which mediated salt-stress responses in *Arabidopsis* [19]. However, the research on SR proteins is lacking in cotton.

The genus *Gossypium* contains two cultivated tetraploids species, *G. hirsutum L.* (AD1) and *G. barbadense L.* (AD2), which originated from transoceanic hybridization of an A-genome-like species, *G. arboreum* with an A-genome-like species, and *G. raimondii* (D5) with a D-genome-like species at around 1~1.5 million years ago (MYA) [20]. In addition, as a significant source of natural fibers for the textile industry, cotton has played an essential role in the global economy [21]. However, various abiotic stresses affect the growth and yield of cotton and other plants [22,23]. Thus, it has become an indispensable scientific issue to identify and screen genes with multiple stress resistance functions. Moreover, genome sequencing has achieved remarkable results in cotton currently, making it possible to systematically identify and study gene families in cotton [20,24,25,26].

In this study, for the first time, we systematically identified SR genes in four *Gossypium* species and performed phylogenetic, gene structure, motif prediction, duplication events, selective pressure, and *cis*-elements analysis. In addition, the spatial expression patterns of *GhSR* genes and their response to NaCl and PEG600 treatments were also monitored. The WGCNA analysis showed that *GhSCL-8* co-expressed with many transcription factors. The Y2H assays indicated that *GhSCL-8* interacts with the *GhSRs* from other subfamilies. Next, we performed subcellular localization analysis of *GhSCL-8*, which is expressed in the nucleus. Finally, we found *GhSCL-8* silenced plants were more sensitive to salt stress than control plants. This study laid a foundation for a comprehensive understanding of the *SR* family and revealed the vital role of *GhSCL-8* in plant salt tolerance. This work will offer a comprehensive view of SR proteins in cotton and improve cotton breeding under salt tolerance.

## 2. Results

### 2.1. Identification of SR Proteins

A total of 29, 29, 56, and 55 SR genes were identified in *G. arboretum* (A_2_, 2n = 2x = 26), *G. raimondii* (D_5_, 2n = 2x = 26), *G. barbadense* (AD)_2_, 2n = 4x = 52), and *G. hirsutum* (AD)_1_, 2n = 4x = 52), respectively (Table S1). The number of SR proteins in diploid *Gossypium* species is larger than that of *Arabidopsis* (18 SR genes) and *O. sativa* (24 SR genes). Meanwhile, the number of SR proteins in each allotetraploid is the sum of the two diploid species. The 169 predicted cotton SR proteins ranged from 160 to 372 amino acids (aa) in length with an average of 249.9 aa, the molecular weight varied from 17.68 to 93.28 kDa, and the theoretical isoelectric point (pI) ranged from 6.24 to 11.99. The grand average of hydropathy (GRAVY) in this family showed that all proteins had negative values (−1.82~−0.43), indicating that all SR proteins were hydrophilic in cotton (Table S3). The average values of the above physical and chemical characteristics in allotetraploid cotton and their diploid progenitors were approximately equal to each other.

### 2.2. Phylogenetic Analysis and Classification of SR Proteins

**Figure 1:**
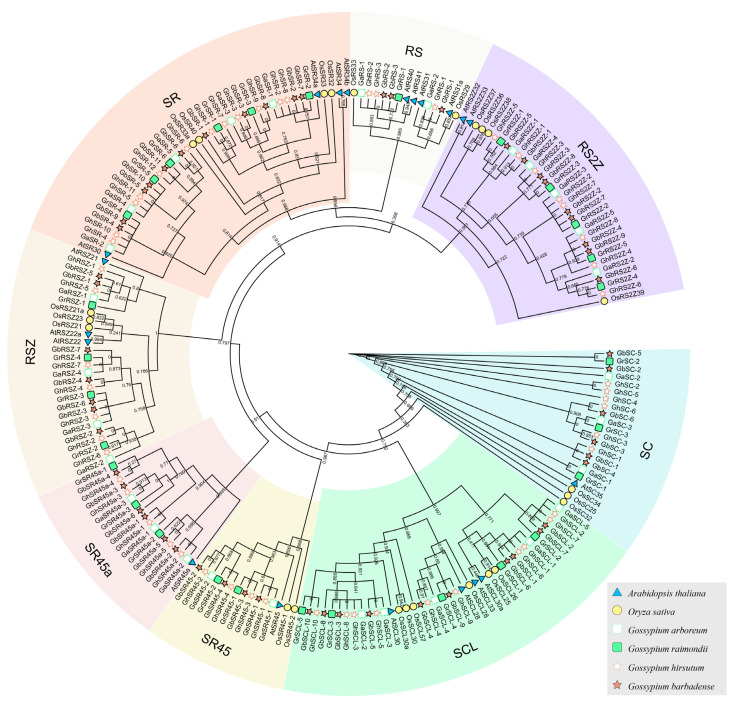
Phylogenetic analysis of predicted SR proteins from six plant species. Phylogenetic analysis grouped all 223 SR proteins into eight subfamilies in a tree calculated using the FastTree. Each subfamily was shown in different color. The prefixes At, Os, Ga, Gr, Gh, and Gb are used to identify SR proteins from *A. thaliana*, *O. sativa*, *G. arboreum*, *G. hirsutum*, *G. raimondii* and *G. barbadense*, respectively. In addition, At and Dt refer to the A and D sub-genomes of *G. hirsutum* and *G. barbadense*, respectively.

Together with the AtSR and OsSR proteins from the previous study, a phylogenetic tree including 209 SR proteins was generated. As clearly shown in the phylogenetic tree, these SR proteins can be divided into eight distinct subfamilies: SR, RS, SC, SCL, RSZ, RS2Z, SR45, and SR45a. No species-specific subfamily was found in cotton species (Figure 1). The members in the SR subfamily constituted the largest number (27.81%), followed by the SCL (20.11%) and RS2Z (17.75%) subfamily, whereas the RS subfamily had the lowest number (5.33%) (Figure 1 and Table S3). The member of each subfamily from the *G. hirsutum* and *G. barbadense* cotton was almost twice that from the diploid cotton.

**Figure 2:**
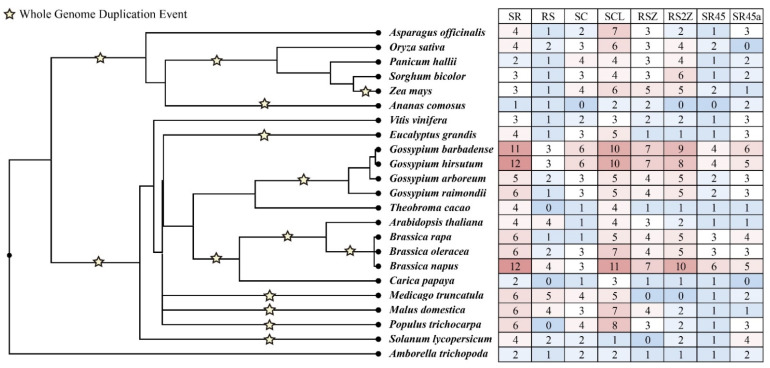
The phylogeny of the 23 plants analyzed in this study and the number of SR proteins identified in each subfamily. The order of tree branches and divergence time are derived from the TimeTree database (http://timetree.org/) (accessed on 17 August 2021).

To explore the evolution pattern of SR proteins, we further identified the SR protein in other 17 species and deployed the same phylogenetic analysis. In most chosen species, as with Arabidopsis and cotton, the SR proteins were classified into eight subfamilies, and no species-specific subfamily was found. It seems that the expansion of SR genes in most plants was affected by WGD events. For instance, the diploid cotton’s SC, SCL, and RS2Z subfamilies are 3~5 times larger than *T. cacao*. In addition, in *Gossypium* and *Brassica* species, we also find that allotetraploidization events play a role in expanding SR genes. The members of allotetraploid species were roughly equal to the sum of their diploid progenitors in most subfamilies. The SR and SCL subfamilies contained a large number of SRs in most species. However, some subfamilies cannot be detected in many species; *A. comosus* lost SC, RS2Z, and SR45 subfamilies; the RS and SR45a subfamilies were not detected in *C. papaya*; *M. truncatula* does not own the members of RSZ and RS2Z subfamilies (Figure 2). These results might suggest that most SR genes were conserved in the evolution progress, whereas few SR subfamilies were lost in many plant species.

### 2.3. Gene Structure and Conserved Motifs among Four Gossypium Species

The domain analysis showed that all SR proteins contain one or two RRM_1 domains located in the N-terminal in most cases. Besides the RRM_1 domain, one and two zf-CCH domains were found in the RSZ and RS2Z subfamily members, respectively. The RRM_1 domain is approximately located in the middle part of the SR45s. Interestingly, the RRM_1 domain of *GaRS-2* is located at the C-terminal, and the N-terminal of GaRS-2 contains PPR, PPR_1, and PPR_2 domains. GaSC-3 owns an OCRE domain in the C-terminal (Figure S1).

**Figure 3:**
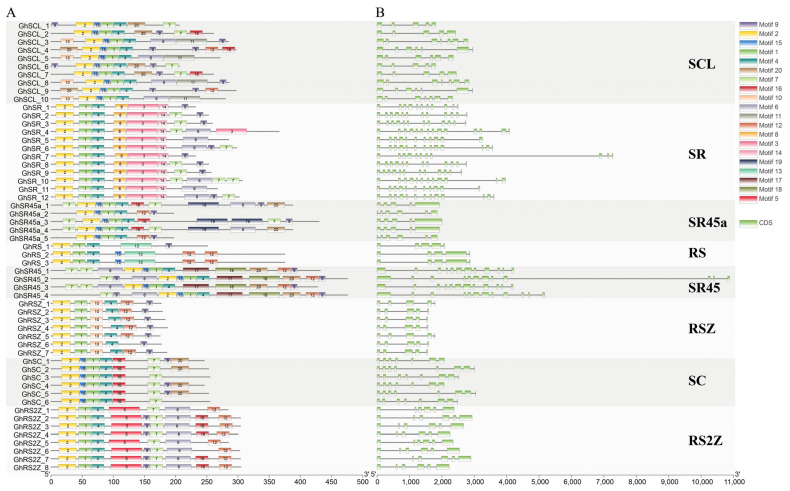
Gene structures and conserved motifs of GhSR genes. (**A**) Distribution of conserved motifs in SR proteins. Colored boxes indicate 20 putative motifs. (**B**) Exon/intron organization of SR genes. Green boxes represent exons, and black lines represent introns.

A total of 20 motifs were detected in *GhSR* proteins, and the motif pattern was conserved in each subfamily. Motif 1 and motif 2, identified as RRM_1 domain, combined with motif 4, distributed in every SR protein. Motif 3 and motif 13, also identified as RRM_1 domain, were mainly found in SR and RS subfamily. Motif 5 was identified as zf-CCHC domain, mainly distributed in the RS2Z subfamily. Motif 14, 19 was only found in SR and SR45a subfamily, respectively. In addition, motif 17 and motif 18 belong exclusively to the SR45 subfamily (Figure 3, Table S4). We also found similar results in GaSR, GrSR, and GbSR proteins. (Figure S2, Table S4). The structural differences between different groups and conserved motifs within the same and different groups indicated functional diversity of the SR gene family in *G. hirsutum*.

The gene structure (exon and intron) of SR genes was relatively conserved in RSZ (5~6 exons), RS2Z (6~7 exons), SR45 (12 exons), and SR45a (5~7 exons) subfamilies, whereas the number of exons in the members of SR (11~15 exons), SC (7~15 exons) subfamilies varies dramatically (Figure 3 and Figure S2). These results suggest that the gene structure and protein architecture of cotton SR proteins are dramatically conserved within each specific subfamily. Phylogenetic analysis indicated that the relationship between classification and evolution was consistent.

### 2.4. Chromosome Location, Gene Duplication and Selection Pressure of SR Genes

**Figure 4:**
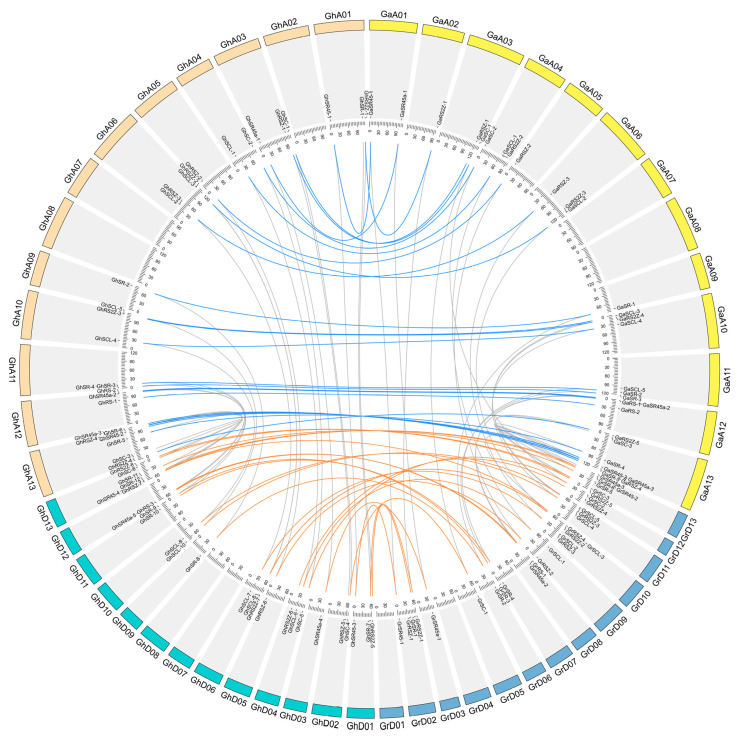
The chromosome distribution and collinearity analyses of GhSR genes. The SR genes of *G. hirsutum* locate on the 21 chromosomes. GaA01-GaA13 and GrD01-GrD13 correspond to the chromosome of *G. arboreum* and *G. raimondii*, respectively; GhA01–GhA13 and GhD01-GhD13 represent chromosomes of the At sub-genome and the Dt sub-genome of *G. hirsutum*. The collinearity gene pairs between *G. hirsutum* and the diploid species are linked by blue and orange lines.

For the two diploid cotton species, the SR genes are located on the 11 chromosomes. The A07, A08, D01, and D04 lack SR genes. For *G. hirsutum*, the SR genes are located in 10~11 chromosomes in each subgenome and lack in GhA02, GhA07, GhA08, GhD07, and GhD08. Therefore, after the allotetraploidization event, the location of some GhSR genes might be altered, and some chromosomes lost the SR genes. GhA05, GhA09, GhD03, GhD05, and GhD09 had only one GhSR gene, whereas GhAt13 and GhDt13 had the most SR genes in At-subgenomes and Dt-subgenomes, respectively. Therefore, the distribution of GhSR genes among 21 chromosomes is imbalanced (Figure 4, Table S3).

Three duplication types were detected in cotton’s SR genes, including WGD, dispersed, and transposed duplication events. The WGD and dispersed duplication were detected in all subgenomes, whereas the transposed duplication events were only found in *G. arboretum*. Apparently, the WGD event was the major force in the SR gene’s expansion, followed by tandem duplication (Table S4). For *G. hirsutum*, the SR genes were conserved during the allotetraploidization event, whereas four SR genes might have been lost in the allotetraploidization event. Moreover, the number of SR genes in *G. hirsutum* is not biased between A-subgenomes and D-subgenomes (Figure 4).

We also analyzed the Ka/Ks ratio of SR genes within each diploid cotton species and each subgenome of allotetraploid species. All the Ka/Ks ratios of gene pairs were lower than 1, which indicated that all the SR genes might experience strong purifying selection pressure during evolution and suggests that the protein functions may be conserved after the expansion (Table S5).

### 2.5. The Analysis of Cis-Elements in GhSRs’ Promoter

The *cis*-elements in the 2000 bp upstream sequence of the GhSRs were predicted in PlantCARE. We detected the hormone-responsive and stress-related *cis*-elements, which mainly included ABA-responsive (ABRE), MeJA responsiveness (CGTCA-motif), gibberellin-responsive (GARE), ethylene-responsive (ERE), salicylic acid-responsive (TCA), and auxin-responsive (TGA) elements. The *cis*-elements responding to environmental stress were also detected, including anaerobic induction (ARE), low-temperature-responsive (LTR), drought inducibility (MBS), *cis*-element related to dehydration (DRE), and defense/stress-responsive (TC-rich) elements. The ERE, ARE, and ABRE elements were found in the most upstream sequence of SRs (Figure S6). These results suggested that either abiotic stress or stress-related hormones could regulate the expression of these SR genes.

### 2.6. The Expression Analysis of SR Proteins

**Figure 5:**
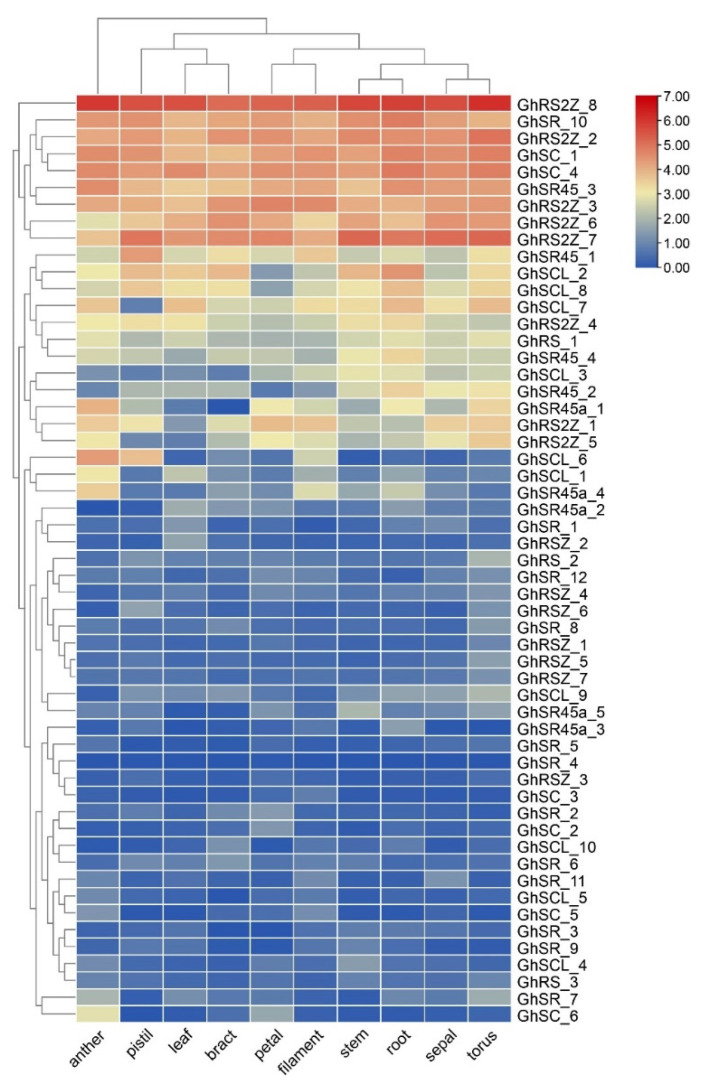
Expression heatmap of GhSR genes in different tissues and organs. The colors varied from blue to red represent the scales of the relative expression levels.

To investigate the differences in the expression of the *GhSRs*, we analyzed the expression profiles of the *GhSRs* in different tissues based on RNA-seq data. The expression of *SR* genes in 10 different tissues was investigated (Figure 5). The results showed that the expression levels of the *GhSRs* presented significant variability in different tissues. The expression of some genes was higher in most tissues, such as *GhRS2Z_8*, *GhSR_10*, and *GhRS2Z_2*, whereas others showed lower RNA transcript levels in most tissues, such as *GhSR_4*, *GhRSZ_3*, and *GhSC_3*. Some genes showed higher RNA transcript levels only in specific tissues; for example, the transcript level of *GhSCL_6* was highest in the anther and pistil.

**Figure 6:**
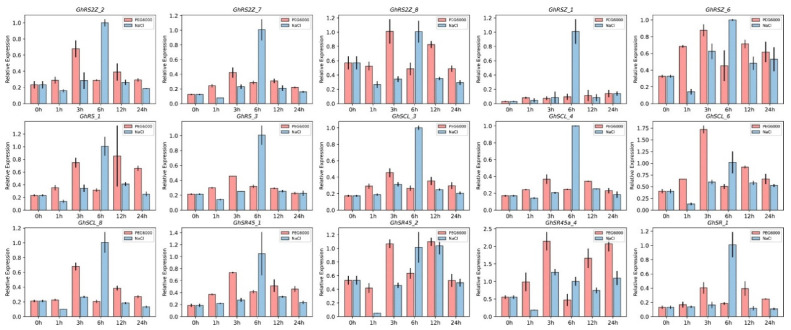
Expression patterns based on qRT-PCR of 15 selected GhSR genes under drought and salt stresses. The red and blue column represents the expression levels of GhSR genes under PEG6000 and salt treatment for 0, 1, 3, 6, 12, and 24 h, respectively.

The biological function of a specific gene is closely related to the spatiotemporal expression of the transcript. Thus, we performed qRT–PCR analysis to investigate the responses of *GhSRs* at 0, 2, 4, 6, 8, 10, 12, and 24 h after NaCl and PEG6000 treatments. Some *GhSRs* were upregulated after NaCl and PEG6000 treatments at specific time points. For instance, *GhRSZ_1* and *GhSCL_4* were significantly upregulated after 6 h salt treatment, and *GhSCL_8* and *Gh_SR45_1* were upregulated after 3 h PEG6000 treatment and 6 h salt treatment (Figure 6). These results indicate that *GhSRs* could be involved in salt and drought stress tolerance in plants.

### 2.7. WGCNA Analysis and Y2H Assays

**Figure 7:**
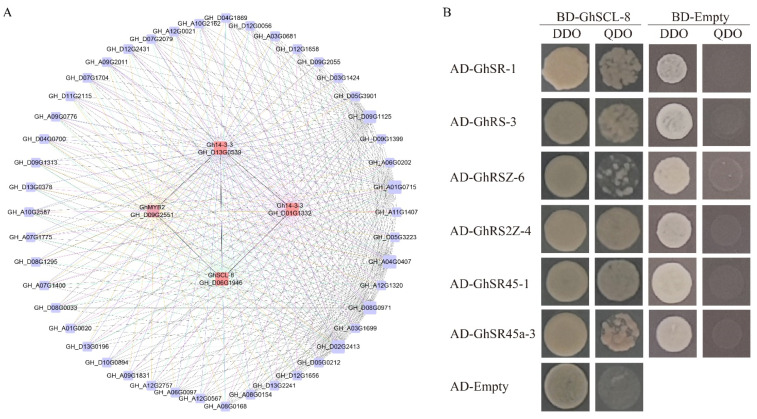
Correlation analysis among *GhSCL-8* and related genes. (**A**) The correlation network of *GhSCL-8* in salt stresses. All the gene networks are constructed by the weighted gene co-expression network analysis (WGCNA) in which each node represents a gene. (**B**) Two-hybrid screening between *GhSCL-8* and GhSR proteins. The empty pGADT7 (AD) and pGBKT7 (BD) were used as negative controls. DDO represents SD-Leu-Trp plates. QDO represents SD-Leu-Trp-His-Ade plates.

To further explore the function of GhSR genes, we used the RNA-seq data in the previous study to construct WGCNA networks. Then, we found a network related to salt stress contained *GhSCL_8*, which suggests that *GhSCL_8* might have co-expression with *Gh14-3-3*, *GhMYB2*, and other genes to respond to salt stress (Figure 7A).

We next performed Y2H assays to explore the proteins that may interact with *GhSCL_8*. We use AD-empty and BD-empty transformed with pGBKT7-*GhSCL_8*, pGADT7-*GhSR45-1*, pGADT7-*GhRSZ-6*, pGADT7-*GhSR-1*, pGADT7-*GhSR45a-3*, pGADT7-*GhRS-3*, and pGADT7-*RS2Z-4* into yeast cells. They grew on yeast medium DDO (SD/-Leu-Trp) but did not grow on QDO (SD/-Leu-Trp-His-Ade), which suggests that no self-activation was detected. Then, BD-GhSCL_8 was used as the bait to transform into yeast cells with the prey proteins. They grow well both on the yeast medium DDO and QDO, demonstrating that *GhSCL_8* could interact with *GhSR45-1*, *GhRSZ-6*, *GhSR-1*, *GhSR45a-3*, *GhRS-3*, and *RS2Z-4* (Figure 7B). Thus, *GhSCL_8* might interact with other SR genes to regulate the biological process.

### 2.8. Subcellular Localization of GhSCL-8 

**Figure 8:**
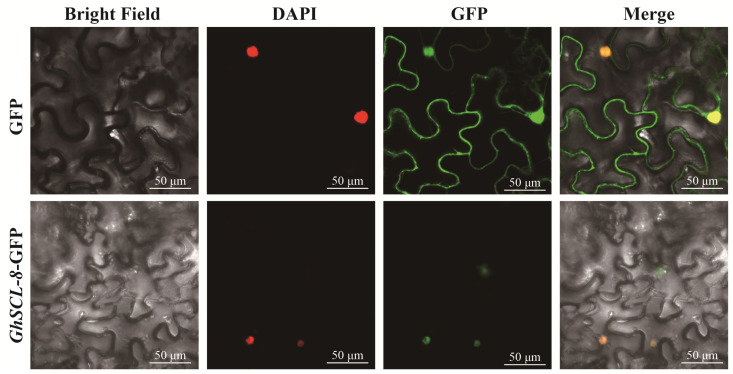
Subcellular localization of the fused *GhSCL-8*-GFP in tobacco leaf cells. The 121-*GFP* was use as the control. Nuclear DAPI staining were expressed in the same cell.

To investigate the subcellular localization of *GhSCL-8*, we use confocal imaging microscopy to analyze the subcellular localization of *GhSCL-8* with the pBI121-*GFP*. Our result showed that GFP expressed from the empty expression vector (pBI121-*GFP*), which served as a control, was mainly distributed in the chloroplast and nucleus, whereas the GhSCL-8-GFP signal coincides with the nucleus-specific DAPI staining, indicating that *GhSCL-8* protein was located in the cell nucleus (Figure 8).

### 2.9. Silencing of GhSCL-8 Attenuates Salt Tolerance in Gossypium Hirsutum

**Figure 9:**
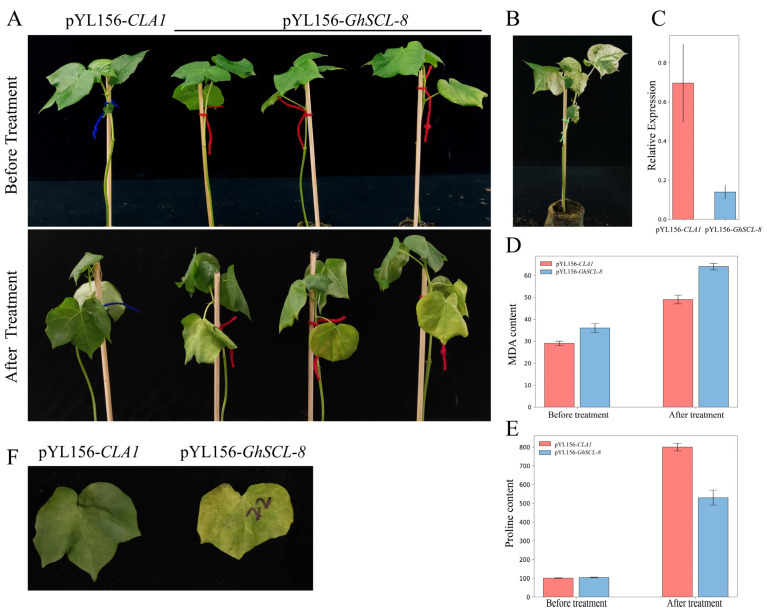
Silencing of *GhSCL-8* reduced salt tolerance of cotton. (**A**) Phenotype of salt-stressed *GhSCL-8*-silenced (pYL156-*GhSCL-8*) and control (pYL156-*CLA1*) cotton plants. Photographs were taken before treatment and 4 days after NaCl treatment, respectively. (**B**) pYL156-*PDS* as the indicator to evaluate VIGS. (**C**) The expression level of *GhSCL-8* in pYL156-*CLA1* and pYL156-*GhSCL-8*) plants. (**D**,**E**) The MDA and proline content of the leaves of pYL156-*CLA1* and pYL156-*GhSCL-8* plants grown under salt treatment. (**F**) The leaves of pYL156-*CLA1* and pYL156-*GhSCL-8* plants after the salt treatment, photographs were taken 4 days after NaCl treatment.

Next, we silenced the *GhSCL-8* gene in cotton via virus-induced gene silencing (VIGS) to analyze its functions under salt stress. After the photobleaching phenotype of infected leaves with pYL156-*GhPDS*, qRT-PCR analysis was performed to detect the gene silencing efficiency in pYL156-*CLA1* and pYL156-*GhSCL-8* plants, the expression level of *GhSCL-8* was significantly lower in pYL156-*GhSCL-8* plants than in control plants (pYL156-*CLA1*) (Figure 9B,C). At the three true-leaf stages, we treated the control and silenced plants with 400 mM NaCl and observed both phenotype after 2 days of NaCl treatment. We found that wilting was more apparent in the leaves of *GhSCL-8* silenced plants than in control plants, and we also found more leaf shrinkage in *GhSCL-8* silenced plants (Figure 9A,F).

The contents of proline and MDA (malondialdehyde) are important indicators used to measure the effects of abiotic stress on plant growth. Proline is a protective agent against osmotic stress, and MDA reflects the degree of lipid oxidative damage [27,28]. We observed that the leaves of pYL156-*GhSCL-8* plants treated with NaCl proline were significantly lower in pYL156-*GhSCL-8* plants than in control plants, and the MDA content in the silencing plants was significantly higher than in the control plants (Figure 9D,E). Therefore, we speculated that the silencing of *GhSCL-8* could decrease the salt tolerance of cotton.

## 3. Discussion

The regulation of AS plays an essential role in various biological processes in the plant, and SR proteins are one of the most critical core regulators in this process. However, the comprehensive study of SR proteins remained elusive in cotton. Here, we explore the characterization of SR proteins in cotton, including the evolutionary relationship, gene expansion, selection pressure during evolution, and expression analysis. In addition, we found the *GhSCL-8* related to a salt stress WGCNA network, and it could interact with GhSRs from other subfamilies. The subcellular localization assays suggest *GhSCL-8* is expressed in the nucleus. Finally, the silencing of *GhSCL-8* could decrease tolerance to salt stress. This study will provide essential information for further investigation of the function of cotton SR proteins.

### 3.1. The Evolution of SR Proteins

In the present study, for the first time, we identified SR proteins in cotton and analyzed their properties. A total of 169 SR genes were identified in different cotton varieties (*G. arboreum*, *G. raimondii*, *G. hirsutum*, and *G. barbadense*). In each allotetraploid cotton species *G. hirsutum* and *G. barbadense*, the number of identified SR genes was almost the sum of the number of SR genes in diploid cotton varieties *G. arboreum* and *G. raimondii*, which strengthens the previous conclusion that the allotetraploid cotton species are originated from the natural hybridization of the two diploid progenitors [20].

Our phylogenetic analysis classified SR protein sequences from the six different plant species into eight major subfamilies. The SR subfamily contains the most serine/arginine-rich family members, whereas the RS subfamily owns the least (Figure 1 and Figure 2). Additionally, the further multiple species analysis showed that no species-specific subfamily was found in this study, whereas some subfamilies were lost in a few cases (Figure 2). Both of the *Brassica* and *Gossypium* species are ideal models to study WGD and allotetraploidization events [29,30]. We found that the SR proteins in *Brassica* species showed a similar expansion pattern. For instance, the members in SR and RS2Z subfamily in diploid *Brassica* and *Gossypium* species are around 1.5~5 times that in *Arabidopsis* and *T. cacao*, then increased by two times in the tetraploid species (Figure 2). Further analysis showed that the expansion of SR genes in cotton is mainly contributed by the WGD event (Table S5). In addition, compared with the diploid species, the most SR genes in allotetraploid species were well conserved in the allotetraploidization events (Figure 4). These results showed that polyploidization events, including WGD and allotetraploidization events, play a significant role in the expansion of the SR gene family.

Functional differentiation might occur in the duplicated genes, including partial or complete loss of the previous functions, gaining new functions, or maintaining the original functions [29,31,32]. The calculated Ka/Ks values of paralogous gene pairs for SR genes in cotton were <1, which suggested that the SR gene family underwent strong purifying selection pressure during evolution (Table S6). Additionally, the purification selection in cotton subjugated the expansion of the SR gene family, reduced the deleterious loss-of-function mutations at duplicated loci, increased fixation, and retained the function of the newly duplicated genes in cotton.

### 3.2. Chromosomal Location, Protein Motifs, and Gene Structure Analysis

A total of 55 GhSR genes were evenly distributed on 21 At and Dt sub-genome chromosomes. A total of 29 out of 44 GhSR genes were located on 12 At chromosomes (A01, A03, A04, A05, A06, A09, A10, A11, A12, and A13), and 26 were located on 11 Dt chromosomes (D01, D02, D03, D04, D05, D06, D09, D10, D11, D12, and D13). However, no GhSR gene was located on A02, A07, A08, D07, and D08 chromosomes. The distribution of GhSR gene in 21 chromosomes is uneven. A13 and D13 chromosomes contain the most SR genes, whereas the A05, A09, D03, D05, and D09 chromosomes own the least. However, the distribution of the GhSR gene in At (29 SR genes) and Dt (26 SR genes) chromosomes is not biased.

The RRM_1 domain was highly conserved in all GhSR proteins, and the zf-CCHC domain was found in RSZ and RS2Z subfamilies (Figure S1). Additionally, we identified 20 motifs in cotton SR proteins. The distribution of SR protein motifs was found slightly different in different subfamilies. However, they were conserved within each subfamily. For example, motif 5 belong exclusively to the RS2Z subfamily, and motif 3 and 13 are only present in SR and RS subfamilies. Motifs 1, 2, and 4 exist in all GhSR proteins, indicating that motifs 1, 2, and 4 are the most conserved in cotton SR proteins (Figure 3). Gene structure is an important feature and is determined by insertion/deletion events [33]. Gene structure analysis demonstrated that SR genes have a different structure in different subfamilies. For example, the intron number in SC (7–15) and SR (11–15) subfamily varies dramatically, whereas the gene structures in RSZ (5–6) and RS2Z (6–7) are relatively conserved (Figure 3). The SR protein function variation might originate from these diverse features.

### 3.3. cis-Elements and Expression Analysis

Promoters contain *cis*-elements that are important for a gene’s specific expression and function during growth and development. Some critical stress and hormone-responsive *cis*-elements for the plant were found in the promoter region of almost all SR genes (Figure S4). Therefore, promoter analysis indicated that the SR genes in cotton play roles in various stress responses.

The biological function of a specific gene is closely related to the spatiotemporal expression of the transcript. Therefore, the expression patterns of GhSR genes were investigated based on available transcriptomic data in various tissues. The transcriptomic data results showed that many GhSR genes, such as *GhRS2Z_8*, *GhSR_10,* and *GhRS2Z_2,* were ubiquitously expressed in all tissues. However, *GhSR_4*, *GhRSZ_3*, and *GhSC_3* showed relatively low expression (Figure 5). Furthermore, qRT-PCR results demonstrated that some GhSR genes showed higher expression patterns in response to abiotic treatments. For instance, the expression of *GhSCL-8* was significantly up-regulated after 6 h salt treatment (Figure 6). The divergence of gene expression in GhSR genes highlighted the extensive involvement of the SR genes in plant development as well as in environmental adaptation in *G. hirsutum*.

### 3.4. GhSCL-8 Might Enhance Salinity Tolerance in Gossypium Hirsutum

Previous studies show that SR genes play significant roles in abiotic stress response. For instance, the heterologous expression of *Populus trichocarpa PtSCL30* and *Manihot esculenta* *MtSCL30A* in *Arabidopsis* could induce hypersensitivity to salt [15,16]. The overexpression of *SR45a* and *OsSR45* could increase the salt tolerance in *Arabidopsis* and rice, respectively [18,19].

In this study, by performing WGCNA analysis, *GhSCL-8* was found in a co-expression network related to salt stress, and this network contained genes that might be related to salt stress in the previous studies [34,35,36] (Figure 7). The Y2H assays showed that *GhSCL-8* could interact with GhSR genes in other subfamilies, including *GhSR45-1*, *GhRSZ-6*, *GhSR-1*, *GhSR45a-3*, *GhRS-3,* and *RS2Z-4*, which were up-regulated in the salt stress. Thus, we speculate *GhSCL-8* might alter the salt stress in cotton, and it is expressed in the nucleus (Figure 8).

Due to factors such as the long time and low efficiency of the cotton transgene, we performed VIGS assays to silence *GhSCL-8* to study its functions because of the short experimental period, simple operation method, low cost, and high efficiency of this method. After the salt treatment, the silencing of *GhSCL-8* in cotton by VIGS wilted more leaves in pYL156-*GhSCL-8* plants than in control plants. Additionally, pYL156-*GhSCL-8* plants possessed higher MDA, whereas the proline contents showed lower content than the control plants (Figure 9). Therefore, we conclude that *GhSCL-8* could play a positive role in salt stress. However, the precise mechanism of the function of *GhSCL-8* requires further study.

## 4. Materials and Methods

### 4.1. The Data Retrieval and Identification of Serine/Arginine-Rich Protein Splicing Factors

The genomes of 23 species and their annotation were chosen in this study (Table S1). The typical and untypical SR proteins of *Arabidopsis thaliana* and *Oryza sativa* were obtained from previous studies [7,18,19]. These SR proteins in model plants were set as the query in a BLASTp search (e-value cutoff = 1 × 10^−10^) to identify the SR proteins in the rest species [37]. The principle to define the SR proteins were according to the previous study: (a) the sequences were further submitted to PfamScan (https://www.ebi.ac.uk/Tools/pfa/pfamscan/) (accessed on 10 August 2021) website to confirm the RRM domain (PF00076) in the N-terminal [38]; (b) the downstream of the RRM domain contains an RS domain at least 50 amino acids and a minimum of 20% RS or SR dipeptides [7]. The protein properties of SR proteins were predicted by the ProtParam module in Biopython [39].

### 4.2. Phylogenetic Analyses

The protein sequences were aligned using MAFFT (v7.310), and BMGE was then used to remove gaps in the alignment with the BLOSUM62 matrix and gap rate cut-off of 50% [40,41]. The aligned protein was then used to construct a phylogenetic tree using FastTree with LG model and finally visualized on the evolview (http://www.evolgenius.info/evolview/) (accessed on 13 August 2021) website [42,43].

### 4.3. Identification of Gene Collinearity and Specific Duplication Events

To analyze gene collinearity, BlastP (E < 1 × 10^−10^, top 5 matches, and m8 format output) and MCScanX (with default parameter) were performed to search all collinearity gene pairs between the different species and subgenomes [37,44]. The duplication event and the related gene pairs within each *Gossypium* species and subgenome was identified and classified by Dupgen_finder (https://github.com/qiao-xin/DupGen_finder) (accessed on 17 August 2021) [45].

### 4.4. The Calculation of Selective Pressure

The detected gene pairs were further aligned by performing MAFFT software and formatted into an AXT format using the ParaAT pipeline [41,46]. Next, the synonymous rate (Ks), nonsynonymous rate (Ka), and their ratio (Ka/Ks) of each gene pair were calculated by Kaks_calculator (v2.0) [47].

### 4.5. The Gene Structure and Conserved Motifs Analyses

The exon/intron position information in *Gossypium* spp. was extracted from the GFF/GTF files. In addition, the full-length protein sequences were submitted to the MEME website (http://meme.sdsc.edu/meme/itro.html) (accessed on 20 August 2021) to detect motifs [48]. The function of each motif was verified in PFAM (https://www.ebi.ac.uk/Tools/hmmer/results/) (accessed on 21 August 2021) and SMART (http://smart.embl-heidelberg.de) (accessed on 22 August 2021) [38,49].

### 4.6. The Identification of cis-Elements

The *cis*-elements in the 2000 bp upstream genomic DNA sequences were submitted to the PlantCARE website (http://bioinformatics.psb.ugent.be/webtools/plantcare/html/) (accessed on 25 August 2021) to predict the *cis*-acting elements [50].

### 4.7. RNA-seq and WGCNA Analysis

The transcriptome data were retrieved from a previous study (Accessions: PRJNA490626), which included ten tissues (the petal, pistil, root, sepal, stem, torus, filament, leaf, anther, and bract) and seedlings treated with salt and drought [20]. To generate clean reads, the raw RNA-seq reads were filtered by Trimmomatic with the default parameter (v0.3.9) [51]. Next, by performing HISAT2 (v2.1.0), the clean reads were mapped to the reference genome (ZJU2.1) to produce SAM (Sequence Alignment/Map) format data and then converted to BAM (Binary Alignment/Map) format data using Samtools (v 1.9) [20,52,53]. The BAM files were assembled into transcripts and generated FPKM (Fragments Per Kilobase of transcript per Million mapped reads) using StringTie (v2.0), and the final expression levels were shown as log2 (FPKM + 1) [54]. The FPKM value was further employed to construct co-expression networks by WGCNA (v 1.69), and the final networks were visualized by Cytoscape software (v3.7.2) (http://www.cytoscape.org/) (accessed on 27 August 2021) [55,56]. 

### 4.8. Plant Cultivation, RNA Isolation, and RT-qPCR Analysis

The sterilized seeds of *G. hirsutum* L. (TM-1) were grown in a mixture of soil vermiculite in an artificial growth chamber, and the environment was adjusted to 16/8 h day/night and 24/16 °C. Three-week-old seedlings were uniformly selected and treated by two kinds of treatment separately: (1) 400 mM PEG; and (2) 400 mM NaCl. Three biological replicates plant samples at 0, 1, 3, 6, 12, and 24 h were freshly collected and frozen in liquid nitrogen and stored at −80 °C till further analyses. The total RNA was isolated using the RNAprep Pure Plant Kit (TIARGEN, Beijing, China) and was treated with DNase I to remove genomic DNA. The RNA quality and purity were measured by a NanoDrop 2000 spectrophotometer (Thermo Scientific, Waltham, MA, USA), and 1 µg of total RNA was used to synthesize first-strand cDNA with the Transcriptor First Strand cDNA Synthesis Kit and oligo-dT primers at 42 °C for 60 min and 72 °C for 10 min. Real-time PCR was performed in a 7500 Fast Real-Time PCR system (Applied Biosystems, Waltham, CA, USA) using SYBR Green Master Mix. Three biological replicates were performed per cDNA sample, and each reaction was prepared in a total volume of 20 µL containing 10 µL of SYBR Green PCR Master Mix, 0.4 µL of each primer, 2 µL (200 ng) of diluted cDNA template, and 7.2 µL of nuclease-free water. The PCR conditions were as follows: 95 °C for 30 s, followed by 45 cycles of 95 °C for 5 s, 60 °C for 15 s, 72 °C for 10 s; and 4 °C to finish. The results were analyzed with the 2^−∆∆Ct^ method [57]. The primers used in RT-qPCR are listed in Table S2.

### 4.9. Yeast Two-Hybrid Assay

The full-length cDNAs of the GhSRs were cloned independently into both the bait vector pGBDK7 and the prey vector pGADT7. The constructs were co-transformed into yeast strain Y2HGold, and the co-transformed yeast colonies were streaked onto SD/-Leu/-Trp DO (DDO) medium. After growth at 30 °C for 72 h, independent colonies of the same size were transferred to SD/-Leu/-Trp/-Ade/-His DO (QDO) medium.

### 4.10. Subcellular Localization

The full-length coding region of *GhSCL-8* was amplified from the *G. hirsutum* variety TM-1 and cloned into the pBI121-*GFP* vectors. The leaves of six-week-old *N.benthamiana* leaves were injected with pBI121-*GFP*, DAPI, and *GhSCL-8*-GFP, respectively. We left the plants in the dark for 24 h after the injection, then light treatment for 48 h. Observations under the laser confocal microscope were recorded.

### 4.11. Virus-Induced Gene Silencing and Stresses Treatment

LMY37, a salt-tolerant upland cotton cultivar, was chosen to perform VIGS assays in this study. A 300 bp fragment (204–503) of *GhSCL-8* ORF was amplified and cloned into the pYL156 vector. The vectors pYL156-*GhSCL-8*, pYL156-*CLA1* (positive control), and pYL192 (helper vector) were transformed into *A. tumefaciens* strain LBA4404. pYL156-*GhSCL-8* and pYL192, pYL156-*CLA1* and pYL192, and pYL156-*GhPDS* and pYL192 were mixed in a 1:1 ratio, then agroinfiltrated into fully unfolded cotton cotyledons [58]. When pYL156-*GhPDS* plants showed phenotype, pYL156-*GhSCL-8* and pYL156-*CLA1* plants were treated with 400 mM salt for 4 days. The contents of MDA and Proline were determined using Malondialdehyde (MDA) Assay Kit and Proline Assay Kit (Solarbio, Beijing, China), respectively.

## 5. Conclusions

SR proteins play an essential role in plant development, growth, and stress resistance. In this study, 169 SR proteins in four cotton species were identified. It was studied from the aspects of phylogenetic analysis, gene structure analysis, transcriptional expression pattern, protein motif analysis, subcellular localization analysis, WGCNA analysis, and so on. We found that the expansion of cotton SR genes was mainly affected by polyploidization events, and they were classified into eight distinct subfamilies. The SR genes were under the purifying selection, the domain, motif distribution, and gene structure of SR proteins were conserved in the evolution. The *cis*-elements and expression analysis demonstrated that SR genes respond to the abiotic stresses. The WGCNA analysis showed that *GhSCL-8* could co-express with many genes responding to salt stress. The Y2H assays indicate *GhSCL-8* could respond to the salt stress with other GhSR genes. The *GhSCL-8* was found located in the nucleus. In addition, we speculate that *GhSCL-8* positively regulated the salt tolerance of cotton by VIGS assays. This study not only provides basic information for the study of SR proteins in cotton but also helps to provide candidate genes to improve the abiotic stress resistance of cotton.

## Data Availability

Not applicable.

## Supplementary Materials

The following supporting information can be downloaded at: https://www.mdpi.com/article/10.3390/ijms23094566/s1.
