# Supplementary figures and images for: A Comprehensive Identification and Function Analysis of Serine/Arginine-Rich (SR) Proteins in Cotton (Gossypium spp.)
Source: Int J Mol Sci. 2022 Apr 20;23(9):4566. doi: 10.3390/ijms23094566 (PMC9105085; doi:10.3390/ijms23094566)

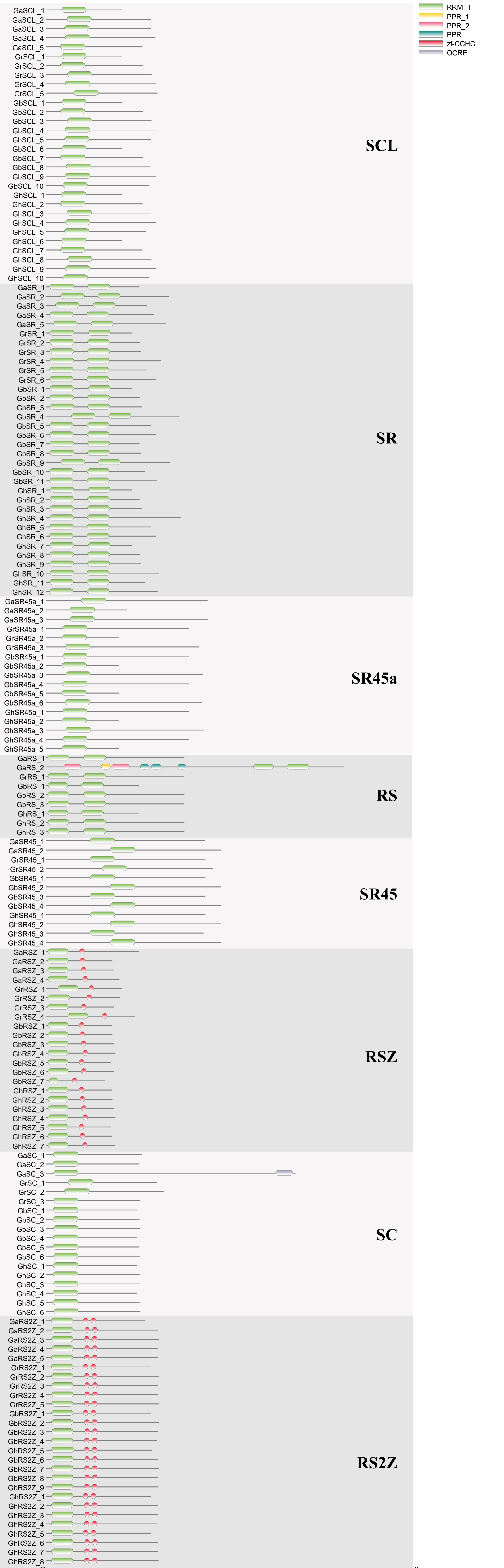

Supplement: Supplementary file 1 [file ijms-23-04566-s001.zip › Figure_S1.pdf]

A

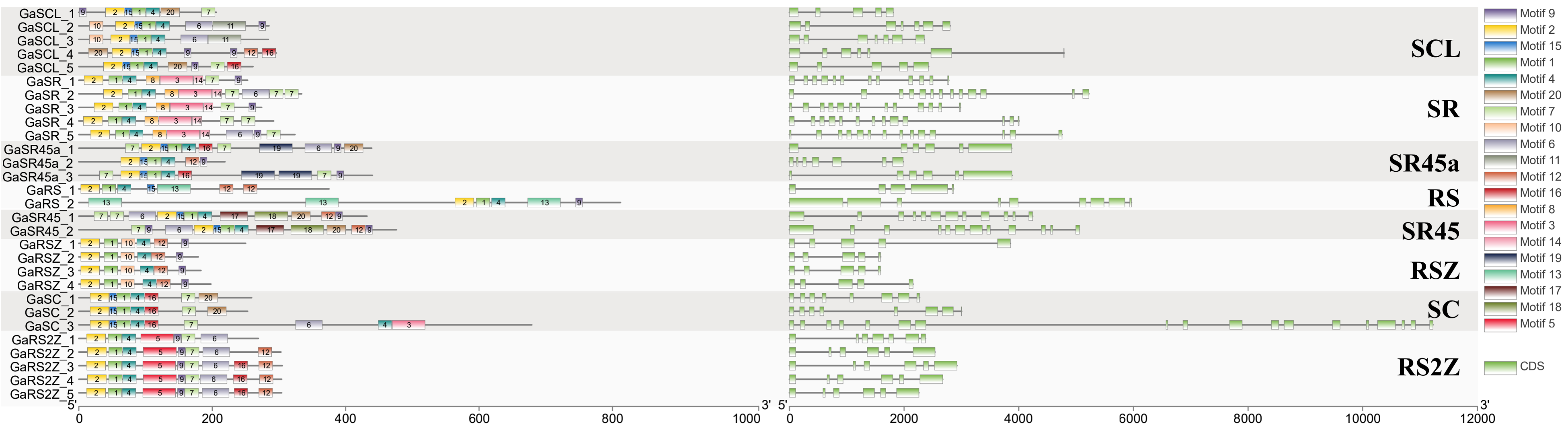

B

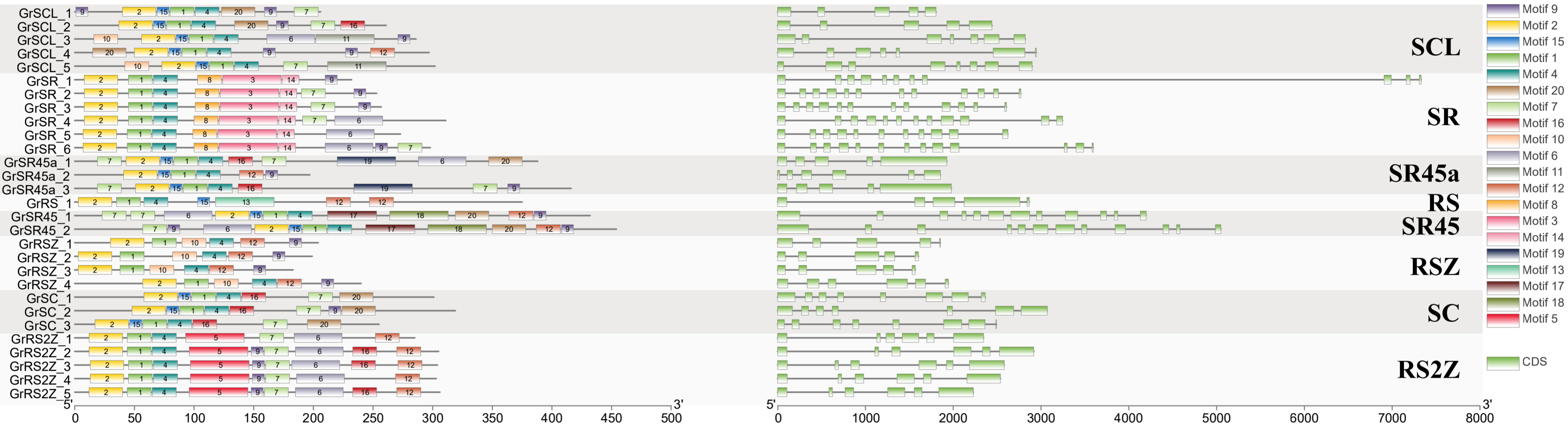

C

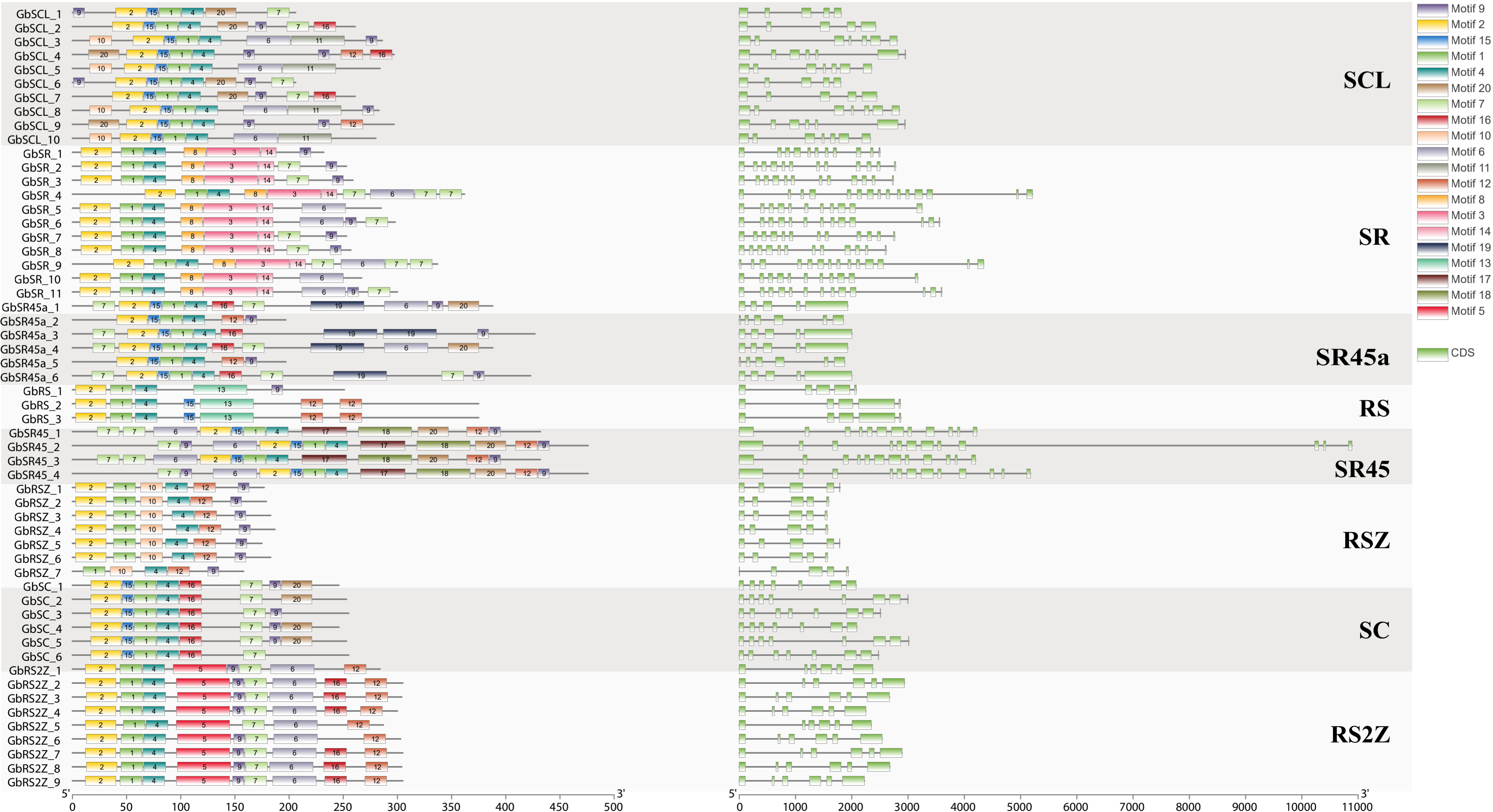

Supplement: Supplementary file 1 [file ijms-23-04566-s001.zip › Figure_S2.pdf]

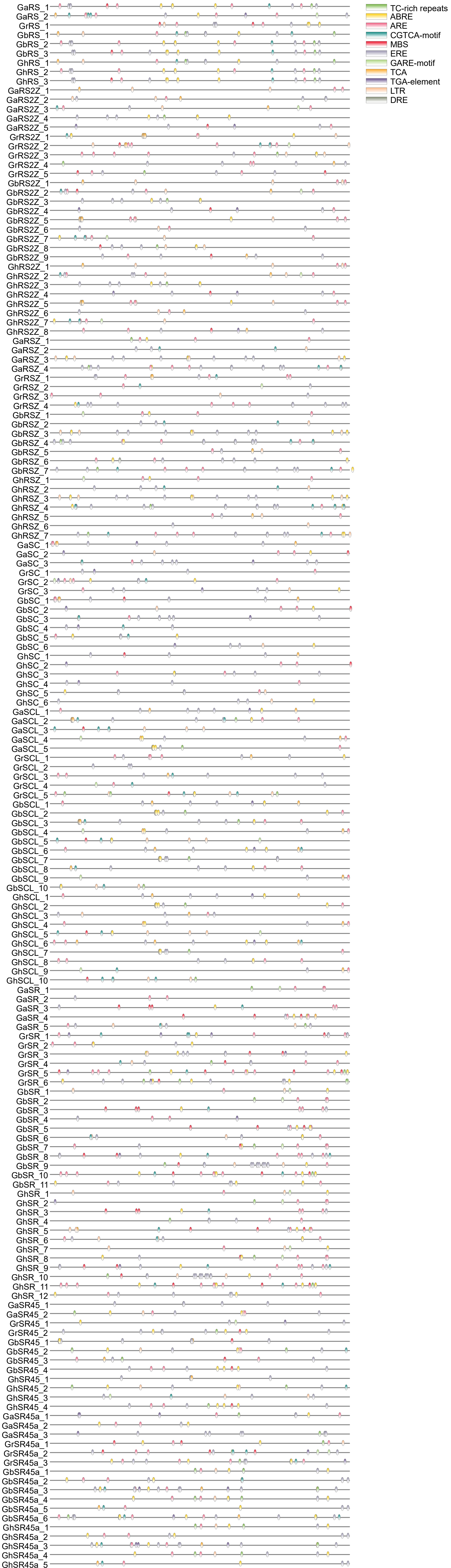

Supplement: Supplementary file 1 [file ijms-23-04566-s001.zip › Figure_S3.pdf]
